# An iPhone Application for Blood Pressure Monitoring via the Oscillometric Finger Pressing Method

**DOI:** 10.1038/s41598-018-31632-x

**Published:** 2018-09-03

**Authors:** Anand Chandrasekhar, Keerthana Natarajan, Mohammad Yavarimanesh, Ramakrishna Mukkamala

**Affiliations:** Department of Electrical and Computer Engineering, Michigan State University, East Lansing, MI 48824 United States

## Abstract

We developed an iPhone X application to measure blood pressure (BP) via the “oscillometric finger pressing method”. The user presses her fingertip on both the front camera and screen to increase the external pressure of the underlying artery, while the application measures the resulting variable-amplitude blood volume oscillations via the camera and applied pressure via the strain gauge array under the screen. The application also visually guides the fingertip placement and actuation and then computes BP from the measurements just like many automatic cuff devices. We tested the application, along with a finger cuff device, against a standard cuff device. The application yielded bias and precision errors of −4.0 and 11.4 mmHg for systolic BP and −9.4 and 9.7 mmHg for diastolic BP (n = 18). These errors were near the finger cuff device errors. This proof-of-concept study surprisingly indicates that cuff-less and calibration-free BP monitoring may be feasible with many existing and forthcoming smartphones.

## Introduction

High blood pressure (BP) is a major, modifiable cardiovascular risk factor^1,2^, yet hypertension awareness and control rates are low^3^. Ubiquitous BP monitoring could improve these rates, but existing devices require inflatable cuffs and thus do not afford such monitoring. While cuff-less BP measurement methods are being widely pursued, many of the methods require calibrations with cuff BP measurements^4,5^.

Recently, we proposed a method for cuff-less and calibration-free BP monitoring via a smartphone^6^. The method represents an extension of the time-honored oscillometric cuff BP measurement principle. The idea is for the user to serve as the actuator (instead of the cuff) by pressing her fingertip against the phone to steadily increase the external pressure of the underlying artery, while the phone, embedded with photo-plethysmography (PPG) and force transducers, serves as the sensor (rather than the cuff) to measure the resulting variable-amplitude blood volume oscillations and applied pressure. The phone also visually guides the finger actuation and then computes BP from the measurements just like a cuff device. We developed a device in the form of a custom PPG-force sensor unit affixed to the back of a smartphone to implement the “oscillometric finger pressing method” and showed that the device can be usable and accurate compared to cuff devices. However, the need for special sensors above and beyond the smartphone limits the accessibility of the method.

Here, we developed a smartphone application that leverages PPG and force sensors already in the phone to implement the oscillometric finger pressing method. We then tested the application against cuff BP measurements for a proof-of-concept demonstration.

## Results

### iPhone Application

**Figure 1:**
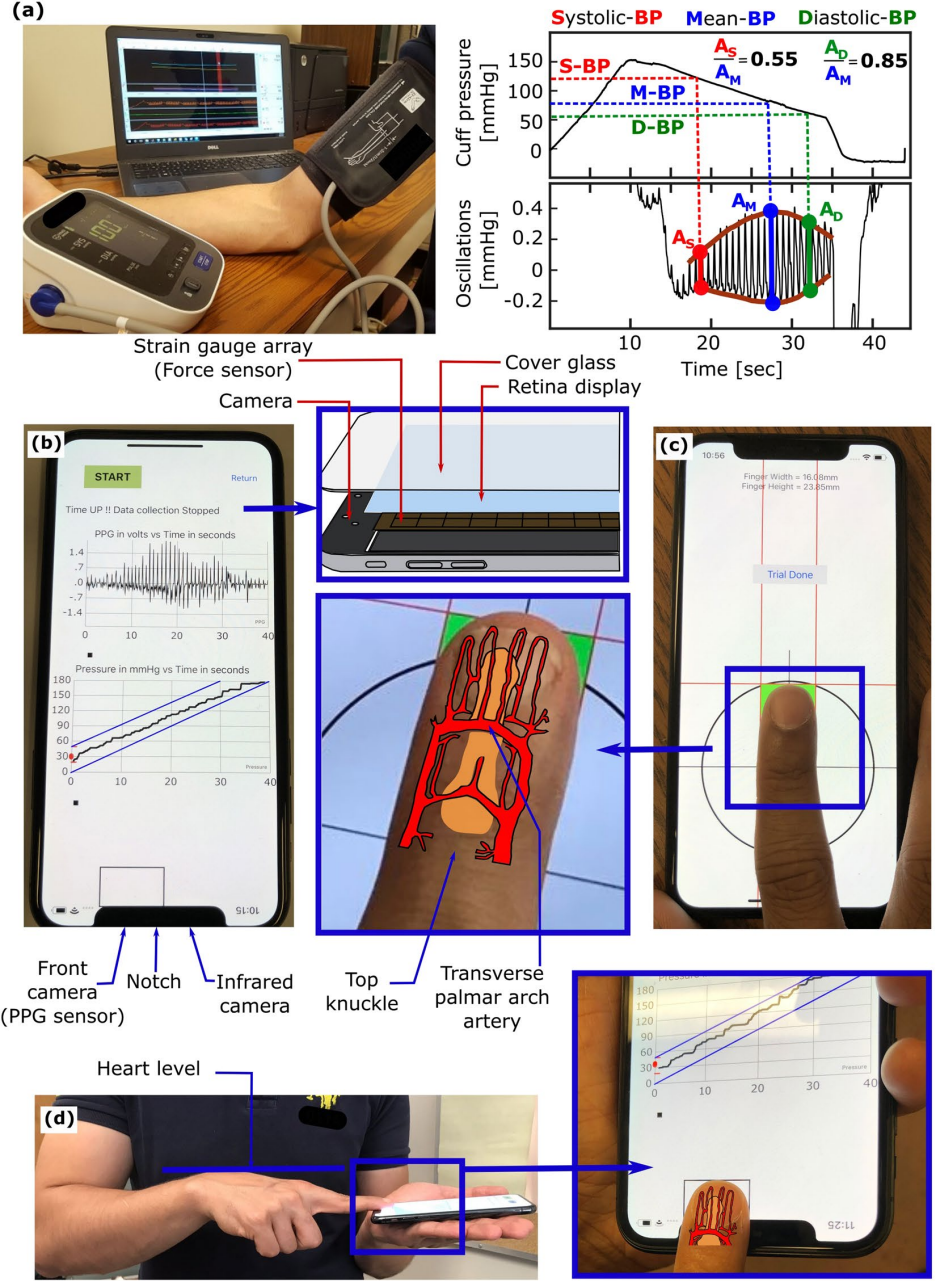
An iPhone application for cuff-less and calibration-free blood pressure (BP) monitoring via extension of the oscillometric cuff measurement principle. (**a**) Photograph of an oscillometric cuff device and diagram of BP computation from the measured cuff pressure and blood volume oscillations. Reproduced from ref.^6^. (**b**) Photograph of an iPhone X application to implement the “oscillometric finger pressing method” by measuring finger pressure via the strain gauge array under the screen and finger blood volume oscillations via the front camera. Insert redrawn from ref.^7^. (**c**) Photograph of a user initializing the application by measuring fingertip width and height from the top of the fingertip to the artery near the middle of the fingertip. (**d**) Photograph of the user making a measurement by placing the fingertip within a rectangular box of the measured width and height; holding the phone horizontally at heart level while resting the fingertip flat on the phone; and pressing to increase the pressure within the two target blue lines.

Figure 1a illustrates the oscillometric cuff BP measurement principle, and Fig. 1b shows the application developed to extend the principle to measure BP with the latest iPhone (X model). The front of this phone is all screen, except for a small notch that includes the camera for taking “selfies”. The application employs this front camera as the PPG sensor where the light source is ambient light and/or screen light (bright setting, which we anecdotally found to suffice in the dark). Spatial averaging followed by band-pass (1.8–4.3 Hz) filtering of the red video channel is applied to extract the blood volume oscillations. The application employs the strain gauge array under the phone screen (but not the notch) for employing “peek and pop” via “3D Touch”^7^ as the force sensor. Apple’s UIKit is used to extract the strain gauge output^8^. Through placement of high density weights on the screen adjacent to the camera, the application derives force (F, grams) from the strain gauge output (V) as F = 443.75 V, where V takes on 400 levels from 0 to 0.83 (firm setting). Like our previous device^6^, the application plots the data as they are recorded to visually guide the finger actuation.

Figure 1c shows that the application also includes measurement of the user fingertip dimensions. One purpose of this measurement is to guide fingertip placement on the screen when measuring BP such that the underlying transverse palmar arch artery (at about the middle of the fingertip) is above the camera. Another purpose is to estimate the finger pressing contact area on the screen, which is needed to compute finger pressure as force divided by area. This measurement need only be made once per user, as finger dimensions and pressing contact area hardly change throughout adulthood^9^. Based on a training dataset comprising index fingertip width and height measurements via the application and reference finger pressing contact area measurements via fingerprinting from 20 subjects, the screen finger pressing contact area (A, mm^2^) is calculated as A = 0.56w·h-5.67, where w and h are specifically the fingertip width at the base of the nail and half the height of the fingertip starting from the crease at the top knuckle minus 2.7 mm (distance from the camera center to the screen edge). Note that the fingerprints were obtained during firm pressing and may thus be valid around the maximum blood volume oscillation regime, which includes mean BP and is mainly used for BP computation^10^. Based on fingerprint dimensions from thousands of subjects^9^ and the force measurement specifications above, we estimate that 95% of people could achieve finger pressure at maximum of >178 mmHg and resolution of <2 mmHg with the application. These specifications are largely congruent with BP measurement.

### Usage

The user initializes the application by placing her index fingertip so that the crease at the top knuckle is aligned with the black horizontal line (Fig. 1c). The user or another person then moves the red vertical and horizontal lines to measure the fingertip width and height (Fig. 1c). The user may then measure BP, as shown in Fig. 1d. The user places her fingertip so that it is tightly encompassed by the rectangular box of width w and height h near the camera when viewing from directly above; holds the phone horizontally at heart level while resting her fingertip flat on the phone for uniform, normal direction force application; and presses to keep the finger pressure within the target blue lines until enough data have been obtained. Using the algorithm employed by our previous device^6^, brachial BP is then computed from the finger measurements or a try-again message is outputted. See supplementary video demonstration.

### Accuracy

**Figure 2:**
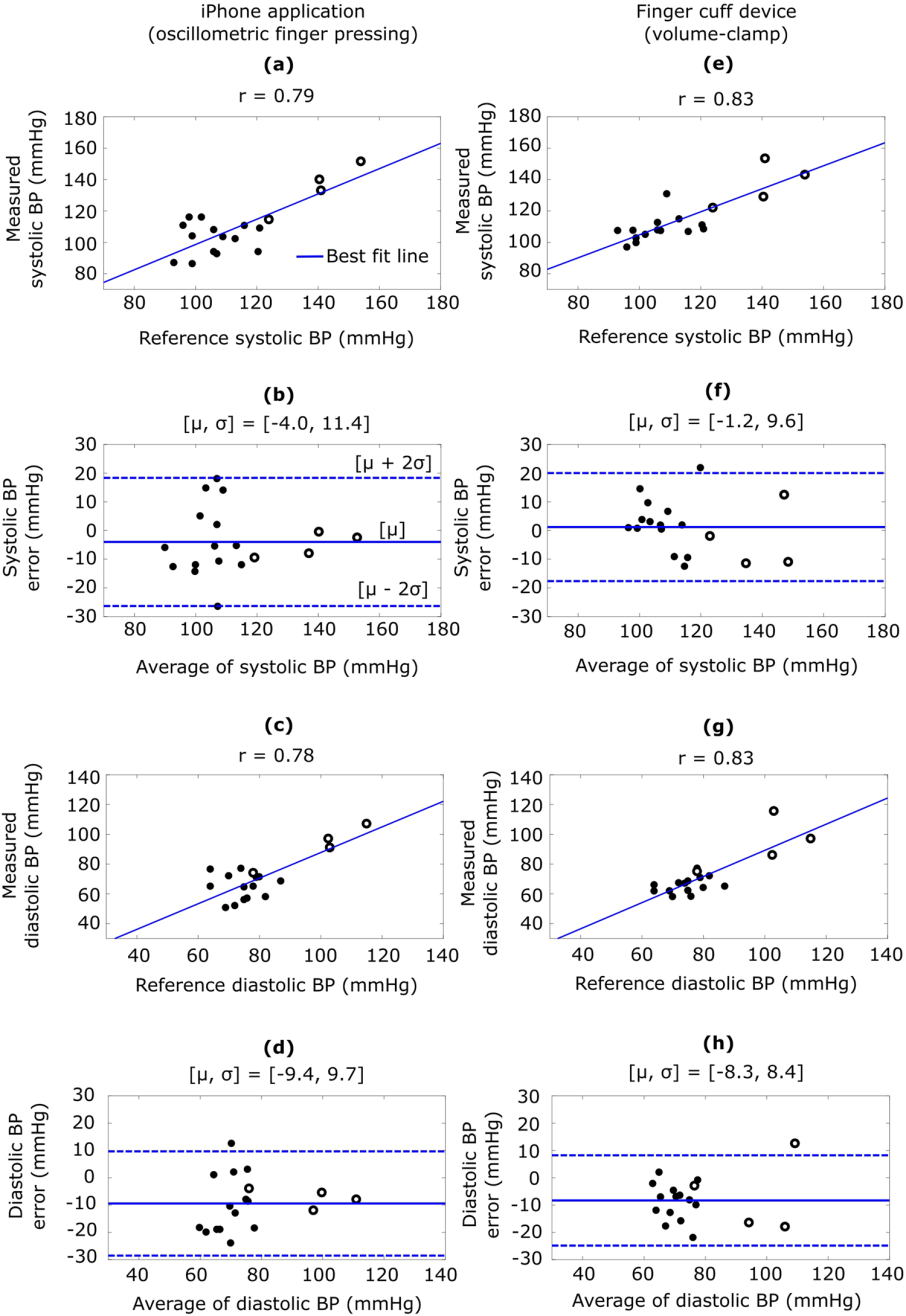
Application accuracy results (n = 18 users). Correlation and Bland-Altman plots comparing the brachial BP measurements from the (**a**–**d**) iPhone application and (**e**–**h**) a finger cuff device to the brachial BP measurements from a standard arm cuff device. The closed circles are data from new users holding the finger devices at heart level, and the open circles are data from experienced users holding the finger devices below the heart to increase BP. r, correlation coefficient; μ, mean of errors (bias error); σ, standard deviation of errors (precision error).

We tested the iPhone application in 20 different subjects. These users were mainly from the cohort employed for testing our previous device (to facilitate comparisons) and included four experienced users of the application. Each new user performed three to six practice trials followed by four measurements. Each experienced user performed two measurements holding the phone well below heart level to raise BP and two normal measurements. The application yielded BP in about half the measurements for the new users and outputted BP in 18 of the users. However, the application did not yield BP in the other two users due to poorly estimated finger pressing contact area (29–43% error relative to fingerprinting compared to <7% mean absolute error in the 18 subjects). The BP measurements from each new user and experienced user holding the device below the heart were averaged and assessed against BP measurements from a standard arm cuff device. Figure 2a–d shows correlation and Bland-Altman plots for the systolic and diastolic BP measurements from the 18 users. The bias errors (µ) and precision errors (σ) of the application were −4.0 and 11.4 mmHg for systolic BP and −9.4 and 9.7 mmHg for diastolic BP over about a 50 mmHg range of BP. Figure 2e–h shows corresponding plots for a finger cuff device, which is FDA-cleared for measuring brachial BP^11^. The application showed errors that were only about 2 mmHg higher on average than the finger cuff device.

## Discussion

The iPhone application errors are close to our previous device^6^. However, the application did not yield BP in two users due to finger pressing contact area mis-estimation, which is not a factor for the device. The application also yielded more try-again messages (about 50 versus 40%) and less repeatable BP measurements (e.g., mean absolute difference between successive measurements at heart level of about 7 versus 5 mmHg) likely due to variability in fingertip positioning despite the rectangular box guide. Hence, not surprisingly, the application may be less effective than our device, which employs application-specific sensors.

However, any reduction in effectiveness may be offset by the increased accessibility of a smartphone application. An estimated 50 million iPhone X models have already been sold^12^. Moreover, other smartphones have 3D Touch capability including iPhone models 6S and higher^7^ and the Huawei Mate S model^13^. Hence, applications for these phones may likewise be developed (with appropriate modifications for differing arrangements of the camera/PPG sensor and screen). In 2017, 328 million iPhones with 3D Touch capability (excluding iPhone 8 and X) were being used^14^. Hence, it is conceivable that the oscillometric finger pressing method could reach about 500 million smartphones already in use.

Our iPhone application should be improved. Most importantly, the finger pressing contact area was mis-estimated in two subjects and variably estimated in some other subjects due to fingertip mis-positioning. In practice, when the application consistently outputs try-again messages or unusual BP measurements, the area could be determined with just one cuff BP measurement (as opposed to periodic cuff calibrations required by competing methods^4,5^). The application could also output a running average of the past several BP measurements (instead of individual BP measurements) to mitigate random variability resulting from fingertip mis-positioning and other factors^5^. In this way, the application may be able to yield BP errors that are closer to the putative bias and precision error limits of 5 and 8 mmHg^15^ than the results reported herein. However, there may be better solutions. One possibility is to measure the area (even at different finger pressures) via the fingerprint sensor under the screen for authentication in upcoming smartphones including the 2019 iPhone X^16,17^. The optimal solution is if Apple were to provide access to an accurate area measurement as the user performs the actuation via the capacitive sensor array also under the screen^7^. Such access may be possible, as superior area assessment may be obtained with Android devices^18^. In addition, the infrared camera also on the notch of the iPhone X (Fig. 1b) for authentication may be used to provide higher-fidelity blood volume oscillations in cold and other low signal conditions^19^. Finally, the BP computation algorithm needs further development to satisfy the accuracy requirements of a regulatory test^15^.

In summary, this proof-of-concept study surprisingly indicates that cuff-less and calibration-free BP monitoring may be feasible with many existing and forthcoming smartphones by leveraging sensors built-in for other purposes. Such ubiquitous BP monitoring may improve hypertension awareness and control rates and thereby help reduce the incidence of cardiovascular disease and mortality.

## Materials and Methods

We performed two sets of human studies under protocols approved by the Michigan State University Institutional Review Board and in accordance with relevant guidelines and regulations. We obtained written, informed consent from each subject. The purpose of the first study was to develop the iPhone application and a method for estimating the finger pressing contact area in particular. The purpose of the second study was to conduct a proof-of-concept evaluation of the application against cuff devices. Note that the application did not output BP or a try-again message in real time for the sake of convenience (as the BP computation algorithm of our previous device was implemented as an Android application^6^ instead of an iOS application needed here). We thus applied the code for the BP computation algorithm of the previous device off-line to the finger measurements from the application while blinded to the cuff BP measurements.

### Application Development

We studied 22 healthy subjects (age, 27 ± 3 years; height, 169 ± 12 cm; weight, 73 ± 11, kg; 45% females). For each subject, we took two or three measurements of the fingertip “rectangular box” width and height (w and h, as defined in the main text) via the application and three fingerprints via an inkpad and graph paper. For each fingerprint, the subject pressed firmly and uniformly in the normal direction. We averaged the w (mm) and h (mm) measurements and computed the reference finger pressing contact area (A, mm^2^) as the average of the number of squares of fingerprint ink on the graph paper that were 2.7 mm above the middle of the fingertip (see rationale in the main text). We plotted A versus each of w, h, and w·h. After excluding two outlier data points, each set of 20 data points appeared to be well fit by a line. We found that A was best predicted from w·h (see line formula presented in the main text).

### Application Testing

#### Experimental Protocol

We studied 20 different subjects (age, 33 ± 10 (18–55) years; height, 169 ± 7 cm; weight, 66 ± 10, kg; 45% females). This number of subjects is congruent with many other published studies on cuff-less BP measurement^4^. We recruited most of these subjects from the cohort employed for testing our previous device^6^. Sixteen of the subjects had never used the iPhone application, whereas the other four subjects were experienced users of the application. The inclusion criteria were: (i) ages 18 to 60 years and (ii) normotensive or hypertensive. The exclusion criteria were (i) cardiovascular disorders other than hypertension or (ii) problems with fine motor control.

We commenced study of each subject by making the same measurements as the first human study. However, in this evaluation study, the application estimated the finger pressing contact area by applying the average of the w and h measurements to the line formula.

For the new users, we then gave demonstrations on how to use the application to make finger measurements. We allowed them to perform three to six practice trials.

We concluded study of each subject with a series of measurements as follows. We obtained three reference BP measurements via a standard oscillometric arm cuff device (BP786, Omron, Japan). We then had the new users make four finger measurements with the iPhone application and the experienced users make two finger measurements while holding the phone well below heart level to raise BP. We next measured the brachial BP waveform with a finger cuff device based on the volume-clamp method (Finometer Model 2, Finapres Medical Systems, The Netherlands). We thereafter obtained two more reference BP measurements using the standard cuff device. We also had the experienced users make two more measurements while holding the phone at heart level at a later time.

#### Data Analysis

We applied the same code employed by our previous device^6^ off-line to compute brachial BP from the entire finger blood volume oscillation and pressure recordings from the application or output a try-again message. We documented the number of BP measurements and try-again messages. We averaged all BP measurements from the iPhone application for each new user and each experienced user holding the device below the heart and averaged the last four BP measurements from the arm cuff device. For the experienced users, we added a ρgh measurement provided by the finger cuff device (where ρ is the known blood density (near that of water), g is gravity, and h is the vertical distance between the heart and device) to the systolic and diastolic BP measurements of the reference arm cuff device. We also likewise measured the reference finger pressing contact area via the fingerprinting.

As in our previous study^6^, we used standard analyses to assess the systolic and diastolic BP measurements from the iPhone application as well as the finger cuff device, each against the reference BP measurements from the arm cuff device. We assessed accuracy qualitatively in terms of correlation and Bland-Altman plots and quantitatively in terms of the correlation coefficient (r), bias error (µ, mean of the errors), and precision error (σ, standard deviation of the errors). We also assessed BP measurement repeatability via the mean absolute difference of successive measurements at heart level per subject in mmHg and evaluated the finger pressing contact area estimates via the mean absolute difference relative to the reference measurements in percent. Note that we only assessed the repeatability of BP measurements made with the iPhone at heart level, as it was not easy to perform BP measurements with the device well below the heart.

## Electronic supplementary material

Video Demonstration of iPhone X Application

## Data Availability

All data and code used in this study may be requested from R.M.
